# Effects of Intergenerational Relationship and Support on mHealth App Adoption Among Older Adults

**DOI:** 10.1093/geroni/igab046.1243

**Published:** 2021-12-17

**Authors:** Yi-Hsuan Tung, Shinyi Wu, Iris Chi

**Affiliations:** 1 University of Southern California, PASADENA, California, United States; 2 University of Southern California, SANTA MONICA, California, United States; 3 University of Southern California, University of Southern California, California, United States

## Abstract

As a growing body of literature examining the effects of mHealth for older adults’ diabetes self-management, how relational factors affect seniors adopting mHealth is still unclear. Guided by the transactional approach of intergenerational relations and the technology acceptance model, this study aims at investigating the perceived ease-of-use, perceived usefulness, and intention-to-use of a mHealth app among older adults with Type-2 diabetes in relation to familial (parent-child) relationship and to e-learning support from child/ren or from external youth volunteering tutors. Using data from the Intergenerational Mobile Technology Opportunities Program (IMTOP), 304 Taiwanese participants (an average age of 64.6 years, 43% female, and 62.5% received at least a high school degree) who had at least a child were included for analysis using structural equation modeling. Results showed that perceived ease-of-use (β = .58, p < .001) and perceived usefulness (β = .27, p < .001) are significant predictors of intention-to-use. Positive associations are found only between external intergenerational, but not familial, e-learning support and perceived ease-of-use (β = .45, p < .001) and perceived usefulness (β = .42, p < .001). Parent-child relationship is positively associated with both familial (β = .73, p < .001) and external intergenerational support for e-learning (β = .36, p < .001), as well as directly (β = .12, p = .030) and indirectly related to intention-to-use. Our findings suggest the importance of intergenerational relationship and appreciation of both familial and external support to facilitate and sustain older adults’ adoption for mHealth programs.

